# Correlation between DNA methylation and chronological age of Moso bamboo (*Phyllostachys heterocycla* var. *pubescens*)

**DOI:** 10.1186/1999-3110-55-4

**Published:** 2014-01-15

**Authors:** Jin-Ling Yuan, Hui-Min Sun, Guang-Ping Guo, Jin-Jun Yue, Xiao-Ping Gu

**Affiliations:** Research Institute of Subtropical Forestry, Chinese Academy of Forestry, Fuyang, Zhejiang China

**Keywords:** Chronological age, DNA methylation, MSAP, *Phyllostachys heterocycla* var. *pubescens*

## Abstract

**Background:**

Chronological age is the primary consideration when studying the physiological development, aging, and flowering of bamboo. However, it’s difficult to determine bamboo’s chronological age if the time of germination is unknown. To investigate the chronological age of bamboo from the genomic DNA methylation profile, methylation-sensitive amplification polymorphism (MSAP) was employed to analyze the genomic DNA methylation of Moso bamboo (*Phyllostachys heterocycla* var. *pubescens*) from stands of nine germination-ages, using six primer pairs which have previously been shown to yield methylation rates that reflect the age of Moso bamboo.

**Results:**

The results showed that the total genomic DNA methylation rates in Moso bamboo at different chronological ages were significantly different, and the increase in genomic DNA methylation rate was consistent with the increase of chronological age. Six primer pairs displayed different genomic DNA methylation rates in Moso bamboo of nine age’s group; however, a significantly positive correlation existed among these primer pairs. An integrated index was obtained by performing principal component analysis on the six primer pairs to represent the genomic DNA methylation levels in Moso bamboo of various chronological ages, and a quadratic curve between the chronological age and genomic DNA methylation levels was obtained.

**Conclusions:**

Such a relationship between DNA methylation and its chronological age may serve a reference for its aging study in Moso bamboo.

**Electronic supplementary material:**

The online version of this article (doi:10.1186/1999-3110-55-4) contains supplementary material, which is available to authorized users.

## Background

The perennial evergreen bamboo is a group of species in Poaceae used for building structures, biomass, and ornamental horticulture as well as panda habitat conservation efforts. Bamboo regenerates by asexual reproduction, thus a stand of bamboo may consist of shoots that emerge at different times but from the same clone/mother plant. Furthermore, bamboo stalks stop widening as they mature, therefore, unlike in trees, the chronological age of bamboo shoots cannot be determined according to annual rings.

The age of a bamboo stand and the age of its individual shoots are two different considerations: 1) the emergence age reflects when an individual bamboo shoot emerged from the ground in a bamboo forest and is used to determine harvesting time; the emergence age can be determined by factors such as skin color of the bamboo stalk; and 2) the chronological age begins when a bamboo seed germinates. The chronological age considers the entire forest from seedling afforestation. Although bamboo culms in a bamboo forest of the same chronological age may emerge from the ground at different times, shoots generally exhibit synchronous developmental progress. For example, synchronous flowering of *Fargesia murieliae* happened across Europe in 1997–1998 after introduced from China, and it was also flowering in the wild in its native range (Shennongjia, China) from 1996–2000 (Gielis et al. 1999; Li and Denich 2004). *F. nitida* began flowering in the early 1990s in the British Isles since its original collection in its native China in 1886, and it flowered subsequently in the mid 1990s to mid 2000s in Europe and North America (Saarela 2007). Chronological age is the primary consideration when studying the physiological development, aging, and flowering of bamboo. However, chronological age is difficult to determine if the time of germination is unknown.

Recent studies have stated that DNA methylation is closely associated with aging, phase changes in the growth and development processes, and age effects of plants (Finnegan and Kovac 2000; Tariq and Paszkowski 2004; Baurens et al. 2004; Demeulemeester et al. 1999; Fraga et al. 2002b; Hasbún et al. 2005). Our previous studies on 5-year-old, 31-year-old and over 60-year-old Moso bamboo showed that significant differences existed in the genomic DNA methylation levels in Moso bamboo at different chronological ages and the levels increase with age (Guo et al. 2011). These results are consistent with those of previous studies in *Pinus radiata* D. Don and *Prunus persica* (L.) Batsch (Fraga et al. 2002a; Bitonti et al. 2002). Importantly, no methylation differences were detected in bamboos within the same chronological age but at different emergence age (Guo et al. 2011). This finding not only verifies that bamboo shoots originating from the same forest stand have the same chronological age, but also indicates that DNA methylation is closely related to the chronological age of bamboo. The objective of this study was to establish a numerical relationship between the chronological age and the DNA methylation of Moso bamboo. This study employed MSAP to analyze the leaf DNA methylation of Moso bamboo from nine chronological ages using six primer pairs selected in our previous studies which showed methylation level differences closely related to the age of Moso bamboo. We anticipate that the findings can serve as a reference for studies on the chronological age of Moso bamboo.

## Methods

### Plant sample

Fresh leaves of Moso bamboo were picked for DNA extraction from eight seeding-afforestation stands with recorded ages (2-, 6-, 7-, 13-, 18-, 32-, 34-, and 44-year-old stands) and a natural stand (with no flowering record for the past 60 years). Those stands were owned by the local forestry center, who gave the permission for the collection of material for the present study. Five bamboo plants in each age group that emerged at the year of studying were selected randomly for samples.

### DNA extraction

An improved CTAB method was used to extract the genomic DNA from Moso bamboo leaves. The purity and concentration of the extracted DNA were detected using a UV spectrophotometer. The DNA quality was evaluated by performing gel electrophoresis using 0.8% agarose gel. The prepared DNA was stored in a -20°C refrigerator for later use.

### Methylation-sensitive amplification polymorphism analysis

The enzyme digestion, ligation, and PCR amplification steps in MSAP analysis were conducted by referencing methods proposed by Xiong et al. (1999) and the reaction system was optimized as required. Aliquots (5 μl) of selected amplification products were mixed with loading buffers of equal volume. After denaturing at 94°C for 10 min, the mixture was cooled in ice for 5 min. Subsequently, the samples were loaded onto a 6% denaturing polyacrylamide gel for vertical gel electrophoresis, followed by silver staining. Finally, the number of amplified bands was statistically analyzed. The primers used in this experiment were the six primer pairs shown to be closely related to Moso bamboo’s chronological age in the study conducted by Guo et al. (2011). These primer pairs are: E3/HM2, E3/HM6, E3/HM7, E4/HM5, E4/HM6, and E5/HM5. See Table 1 for primer sequences.

**Table 1:** **Sequences of adaptors and primers for methylation-sensitive amplification polymorphism analysis**

Adaptors and primers	EcoRI(E)	HpaII/MspI(HM)
Adaptor 1	5*'*-CTCGTAGACTGCGTACC-3*'*	5*'*-GACGATGAGTCCTGAG-3*'*
Adaptor 2	5*'*-AATTGGTACGCAGTC-3*'*	5*'*-CGCTCAGGACTCAT-3*'*
Amplification primer	5*'*-GACTGCGTACCAATTC-3*'*	5*'*-GATGAGTCCTGAGCGG-3*'*
Selective	5*'*-GACTGCGTACCAATTCCA-3*'*(E3)	5*'*-GATGAGTCCTGAGCGGCAC-3*'*(HM2)
Amplification	5*'*-GACTGCGTACCAATTCAG-3*'*(E4)	5*'*-GATGAGTCCTGAGCGGCTA-3*'*(HM5)
Primer	5*'*-GACTGCGTACCAATTCAA-3*'*(E5)	5*'*-GATGAGTCCTGAGCGGCTC-3*'*(HM6)
		5*'*-GATGAGTCCTGAGCGGCTG-3*'*(HM7)

Both *Hpa* II and *Msp* I recognize the same tetranucleotide sequence (5′-CCGG-3′), but exhibit different sensitivities to methylation: *Msp* I cleaves methylated (C/^5m^CGG) and unmethylated (C/CGG) sites of the internal cytosine, whereas *Hpa* II cleaves only the unmethylated site (C/CGG). We used *Hpa* II and *Msp* I isoschizomers (Promega, USA) for double-enzyme cleavage in combination with *EcoR* I, respectively. Each plant sample was analyzed via the two lanes, in which one lane was digested by *EcoR* I/*Hpa* II and the other by *EcoR* I/*Msp* I. Based on presence (marked as 1) or absence (marked as 0) of band, generated MSAP bands could be grouped into four types of methylation patterns. Type I (00): no band present in any lanes. This is attributed to methylation of the external cytosines (on both strands) or a full methylation of both cytosines. Type II (01): bands present in the *EcoR* I/*Msp* I lane, and these MSAP bands were caused by hypomethylation of the outer C relative to the internal C. Type III (10): bands present in the *EcoR* I/*Hpa* II lane, and these bands were associated with hemimethylation of the outer C. Type IV (11): bands present in both two lanes. These bands correspond to unmethylation. The methylation rate obtained by MSAP is generally lower than the sample’s actual methylation level, for the reason that both *Hpa* II and *Msp* I can not cleave sites of the external cytosines (^m^CGG). To make the result more closer with the sample’s actual methylation level, we calculated the methylation rate by the following formula: total rate of methylation (%) = Total number of methylation bands (Type I + Type II + Type III)/Total number of amplified bands (Type II + Type III + Type IV) × 100%.

### Statistical analysis

Statistical analysis of the data, including ANOVA analysis, correlation analysis and principal component analysis were performed using the statistical program SPSS16.0 (SPSS, Chicago, USA). All data were represented by an average of the five replicates (independent plant individuals). If the ANOVA indicated significant results, a Duncan’s mean separation test was then performed (Duncan 1955).

## Results

### Genomic DNA methylation levels in Moso bamboo from stands of different chronological ages

**Figure 1:**
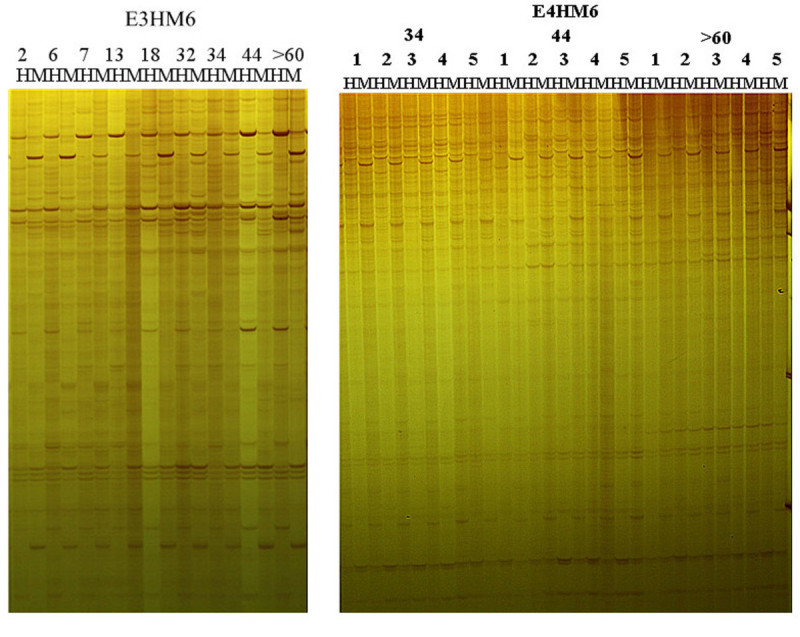
**Partial electropherogram of the MSAP analysis on the DNA methylation in Moso bamboo at different chronological ages.** E3HM6 and E4HM6 represent the primers used for amplifications. Numbers 2 to 60 represent the nine age samples, 2-, 6-, 7-, 13-, 18-, 32-, 34-, 44-, and >60-year-old. Numbers 1 to 5 represent the five repetitions. Each sample has two lanes labeled by H and M, respectively. H represents amplification products of EcoR I/Hpa II enzyme digestion, and M represents amplification products of EcoR I/Msp I enzyme digestion.

The apparent bands obtained from MSAP analysis on the genomic DNA from nine age groups of Moso bamboo using six primer pairs were statistically analyzed. Obvious differences were found in the MSAP bands among different chronological ages, however, few clear difference can be see in the MSAP bands among the five repetitions at the same age. MSAP profiles for Moso bamboo at different chronological ages by primer pairs of E3HM6 and E4HM6 were showed in Figure 1. The total rate of genomic DNA methylation of Moso bamboo from nine ages’ group using six primer pairs were showed in Table 2, the six primer pairs displayed distinct DNA methylation levels in bamboo of different ages. One-way ANOVA was conducted after performing arcsine transformation, the results showed that the total genomic DNA methylation rates in Moso bamboo at different chronological ages were significantly different. *L.S.D* multiple comparison found that variations among the DNA methylation levels in 2-, 6-, and 7-year-old Moso bamboo were insignificant, and it’s also insignificant among those in 32-, 34-, and 44-year-old group. However, significant DNA metylation level variations existed among the groups of 2-year-old to 18-year-old and those of over 60 years-old. These results indicated that more DNA methylation variation exhibited between groups with larger chronological ages distances in Moso bamboo.

**Table 2:** **DNA methylation levels in Moso bamboo at different chronological ages**

Source of variation	Total rate of methylation (%)						Mean	Variance test
Age	E3HM2	E3HM6	E3HM7	E4HM5	E4HM6	E5HM5		
2	14.28	15.38	10.63	17.46	19.4	32.5	18.28 ± 7.58^a*^	SS=0.263, df=8, MS=0.033, F=14.231, Sig=0.000, P<0.01
6	14.28	13.72	15.38	24.24	23.19	32.91	20.62 ± 7.56^a^	
7	12	14.28	21.56	20.31	24.24	30.49	20.48 ± 6.72^a^	
13	14.28	20.41	10.71	20.28	22.39	37.35	20.90 ± 9.18^ab^	
18	18.18	26.41	23.64	23.08	30.77	39.24	26.89 ± 7.33^bc^	
32	25	31.48	25.45	27.69	28.36	44.3	30.38 ± 7.20^bc^	
34	23.07	25.92	28.84	26.56	29.23	36.25	28.31 ± 4.48^bc^	
44	20.83	25	31.48	32.81	31.81	36.25	29.70 ± 5.67^bc^	
63	26.66	50	37.04	32.81	30.76	50	37.88 ± 9.97^cd^	

Total rate of methylation (%) = Total number of methylation bands/Total number of amplified bands × 100%; SS represents Stdev square, df represents degree freedom, MS represents Mean square.

*Values followed by different small letters indicate *L.S.D* significant difference at *p*<0.05.

### Genomic DNA methylation levels in Moso bamboo from nine chronological ages obtained by six primer pairs

**Figure 2:**
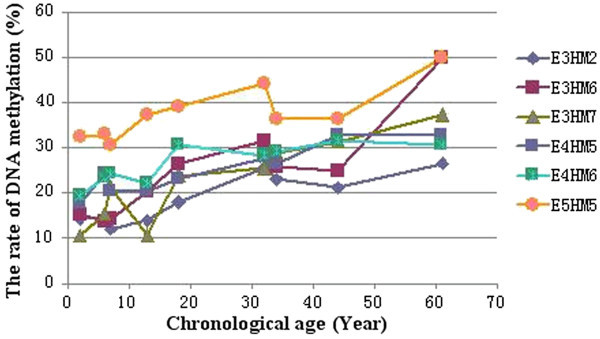
**DNA methylation rate trends for each primer pair across nine chronological ages.** The x axis is the chronological age, the y axis is the DNA methylation rate. Data for E3HM2 are shown with ◆; for E3HM6 with ■; for E3HM7 with ▲, and so on.

Although the genomic DNA methylation were different in the same age by different primer pairs, an overall increasing trend of the total methylation rate can be seen along with the chronological age (Figure 2), which is consistent with the results by 35 primer pair combinations in our earlier study on Moso bamboo of three chronological ages (Guo et al. 2011). We performed correlation analysis on data obtained from each primer pair for all ages and discovered a significantly positive correlation among the six primer pairs, with the greatest correlation coefficient as high as 0.958 (Table 3). This result suggested that the six primer pairs used in this study can effectively represent changes in genomic DNA methylation levels in Moso bamboo in response to the increase in chronological age and that the primers have a relatively high correlation among each other. Therefore, the applicability of these six primer pairs in studying genomic DNA methylation in Moso bamboo is verified.

**Table 3:** Correlation analysis among different primers*

Correlation	E3HM2	E3HM6	E3HM7	E4HM5	E4HM6	E5HM5
E3HM2	1.000	0.880**	0.820**	0.824**	0.763**	0.844**
E3HM6		1.000	0.782**	0.740**	0.685*	0.958**
E3HM7			1.000	0.887**	0.904**	0.630*
E4HM5				1.000	0.835**	0.655*
E4HM6					1.000	0.593*
E5HM5						1.000

*means the significant level of 5% and **means the significant level of 1%.

### Principal component analysis of six primer pairs and determination of the integrated factor

The total genomic DNA methylation rates obtained by the six primer pairs differed within each age group (Table 2). However, the primer pairs showed a significantly positive correlation among each other at each age (Table 3). To transform the complex data of multiple indicators into fewer new indicators, we adopted principal component analysis to identify an integrated factor for clarifying the overall information expressed by the six primer pairs in each age group.

The results of principal component analysis on the six primer pairs (Table 4) showed that the characteristic root of the first principal component was 4.938, thus explaining 82.3% of the total variance. In addition, the characteristic root of the second principal component was 0.672, thus explaining 11.2% of the total variance. The first principal component was the only factor with a characteristic root greater than 1, resulting in its contribution rate of 82.3%. Thus, the first principal component accurately represented the amount of information presented by the overall primer set. Therefore, only the first principal component was adopted as an integrated factor.

**Table 4:** **Statistical data of principal components**

Component	Characteristic root	Contribution rate %	Cumulative contribution rate %
1	4.938	82.294	82.294
2	0.672	11.196	93.490
3	0.168	2.795	96.286
4	0.115	1.909	98.194
5	0.100	1.659	99.854
6	0.009	0.146	100.000

The weightings of each primer pairs on the integrated factor (Principal component 1; Y) were listed in Table 5. The integrated factor, Y, can be expressed as a linear combination of the six primer pairs (x_i,_ i = 1, 2, 3, 4, 5, 6), as in the following equation: Y = 0.191x_1_ + 0.188x_2_ + 0.187x_3_ + 0.184x_4_ + 0.178x_5_ + 0.174x_6_.

**Table 5:** Component score coefficient matrix*

Primers	Principal component 1
1 (E3HM2)	0.191
2 (E3HM6)	0.188
3 (E3HM7)	0.187
4 (E4HM5)	0.184
5 (E4HM6)	0.178
6 (E5HM5)	0.174

*Weightings of each primer pair on Principal component 1.

### Correlation analysis between chronological age and genomic DNA methylation in Moso bamboo

**Figure 3:**
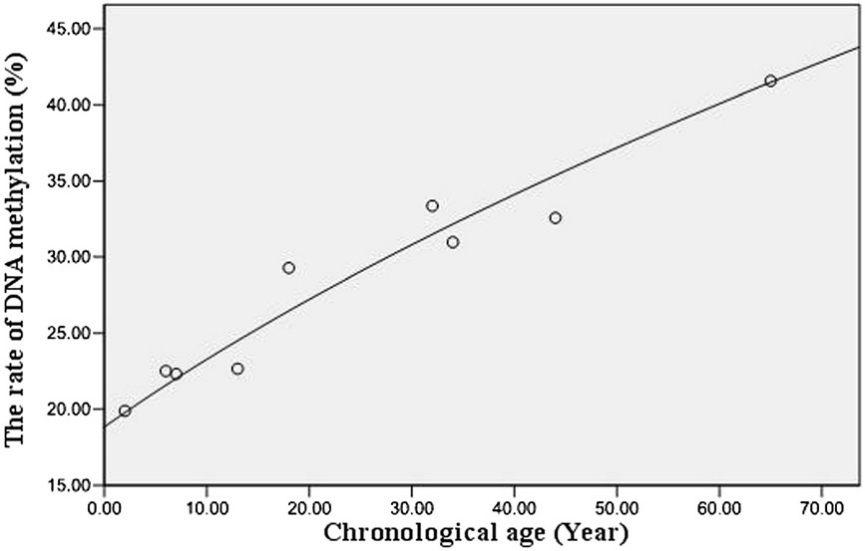
**The relationship between chronological age and DNA methylation level of Moso bamboo.** Using the integrated data from each primer pair, a quadratic curve was fitted using the SPSS software with an equation of Z = 0.034y^2^ + 0.828y - 27.762, where P(Q) < 0.01 and R^2^(Q) = 0.942. Z indicates the chronological age (x axis), y indicates the DNA methylation rate based on the integrated factor (y axis). Data for the nine ages are shown with ○.

The total DNA methylation rate obtained from the six primer pairs were integrated based on the previously described integrated factor equation, it’s found that the DNA methylation levels in Moso bamboo at nine chronological age were of 19.93%, 22.50%, 22.37%, 22.78%, 29.41%, 33.30%, 31.07%, 32.57%, and 41.63%, respectively. The relationship between the chronological age and genomic DNA methylation levels in Moso bamboo was represented graphically (Figure 3). Curve-fitting resulted in a quadratic curve exhibiting a superior fitting effect based on the distribution of the DNA methylation levels in Moso bamboo at different chronological ages. The equation for the quadratic curve is Z = 0.034y^2^ + 0.828y - 27.762, (Z is the chronological age, y is the DNA methylation rate based on the integrated factor), where P(Q) < 0.01 and R^2^(Q) = 0.942. Based on the effectiveness and the coefficient of determination, the quadratic curve can effectively express the numerical relationship between the total genomic DNA methylation level and the chronological age of Moso bamboo.

## Discussion and conclusion

MSAP technology is widely applied in plant genomic DNA methylation studies because of its ease of operation and high sensitivity (Li et al. 2002; Portis et al. 2004; Salmon et al. 2005). Through sequencing and comparative analysis, Cervera et al. (2002) confirmed the effectiveness and reliability of MSAP analysis in a genomic DNA methylation study on *Arabidopsis thaliana*. Selecting appropriate primers for MSAP analysis is advantageous for conducting such a study. In our previous study, six primer pairs which were closely related to the chronological age of Moso bamboo were screened out from thirty-five pairs of primer combinations (Guo et al. 2011). The same six primer pairs were used in this study of genomic DNA methylation in Moso bamboo across nine ages’ group. The results indicated that all six primer pairs stably amplify a large amount of methylated DNA that can be observed as clear and specific bands (Figure 1). The DNA methylation level obtained by each primer pair also increased along with the chronological age in Moso bamboo. Furthermore, there was a significant positive correlation among those six primer pairs. Together, these results demonstrated that those six primer pairs are appropriate for MSAP analysis on genomic DNA in Moso bamboo, and they can stably reflect changes in genomic DNA methylation levels at different chronological ages. This information may serve as a reference for future DNA methylation studies in other bamboo species.

Relevant studies have examined genomic DNA methylation levels and patterns in plants at different developmental stages. For example, the genomic DNA methylation levels were compared in *Pinus radiata D. Don* at mature, juvenile, and juvenile-like stages, and the genomic DNA methylation were examined in *Prunus persica* and *Acacia mangium* during its phase-change developmental stages (Fraga et al. 2002a; Bitonti et al. 2002; Baurens et al. 2004). Similarly, in our study on Moso bamboo at different developmental stages, we found that the genomic DNA methylation level in Moso bamboo is closely related to its aging process (Guo et al. 2011). However, previous studies in plants focused only on genomic DNA methylation levels at different growth and developmental stages, in-depth studies targeting specific ages remains lacked. The latest research on human DNA methylation shows that a predictive model of aging has been built by analyzing the genome-wide methylation profiles of human individuals, aged 19 to 101 (Hannum et al. 2012). We performed MSAP analysis on Moso bamboo to analyze more accurately the changes in genomic DNA methylation levels in Moso bamboo at different chronological ages. We also established a quadratic equation (Z = 0.034y^2^ + 0.828y - 27.762, wherein, R^2^(Q) = 0.942), which expresses the relationship between the genomic DNA methylation level and the chronological age in Moso bamboo. In addition to see in-depth research studies concerning the chronological age of Moso bamboo, this new tool can be practically applied to anticipate the chronological age of Moso bamboo of unknown seeding or in wild stands.

In studies on DNA methylation in rice and corn, functional genes related to stress tolerance and heterosis have been discovered by sequencing differentially-methylated fragments (Hua et al. 2005; Zhao et al. 2007). By examining 3- and 9-year-old Moso bamboo, we also found differently-methylated fragments that exhibit methylation condition changes as bamboo age increases. These fragments may contain methylation variable positions that are closely related to the age of Moso bamboo. Therefore, the next step is to clone these methylation targets and perform southern hybridization to examine their quantitative expression differences in Moso bamboo at different ages. This step may further verify the reliability of methylation analysis results. In addition, sequencing the differentially-methylated fragments may reveal functional genes related to bamboo age in order to investigate the regulatory mechanism of DNA methylation in the physical developmental progress of bamboo.

## Authors’ original submitted files for images

Below are the links to the authors’ original submitted files for images. Authors’ original file for figure 1 Authors’ original file for figure 2 Authors’ original file for figure 3
