# Supplementary figures and images for: Correlation between DNA methylation and chronological age of Moso bamboo (Phyllostachys heterocycla var. pubescens)
Source: Bot Stud. 2014 Jan 15;55:4. doi: 10.1186/1999-3110-55-4 (PMC5432823; doi:10.1186/1999-3110-55-4)

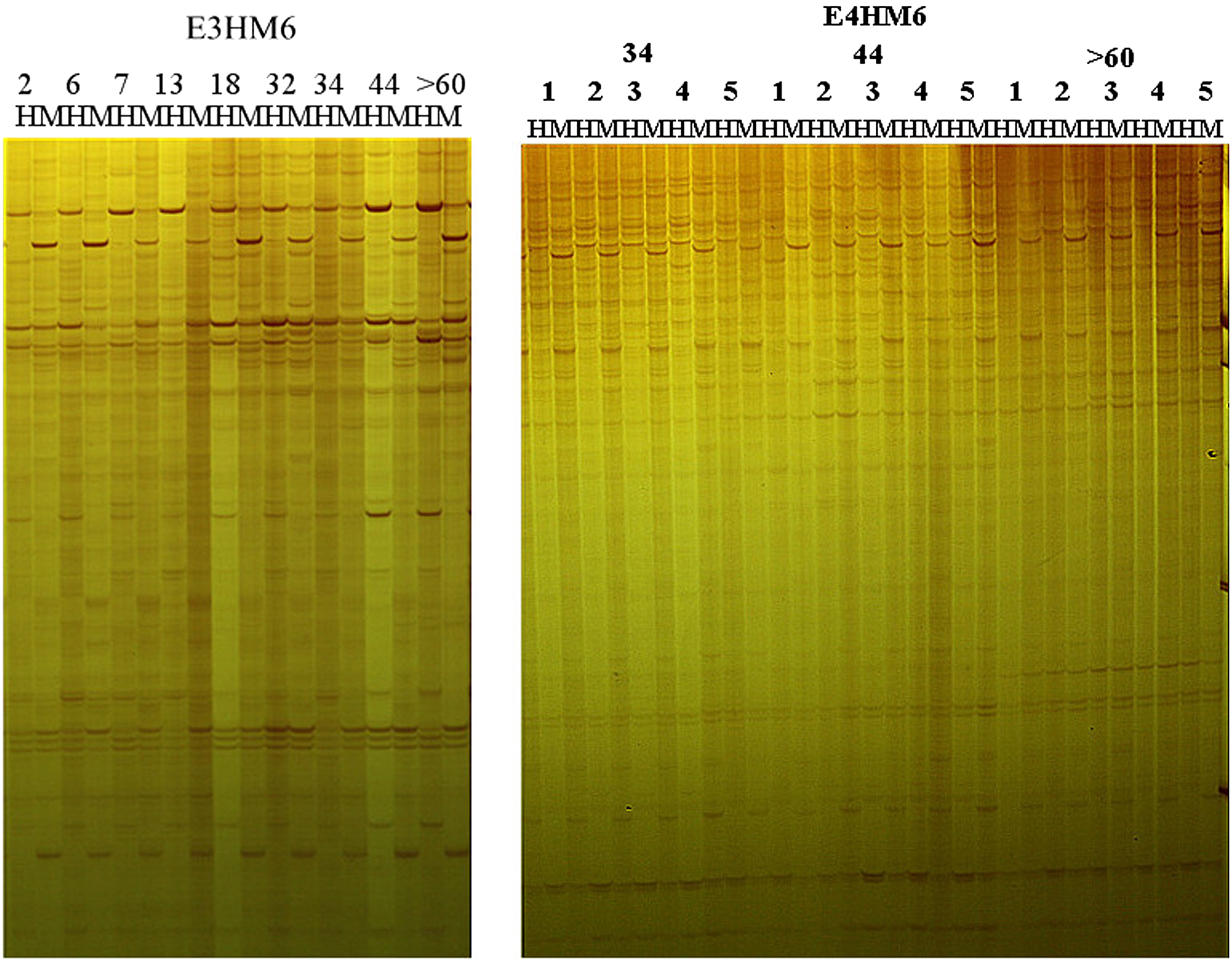

Supplement: Supplementary file 1 — Authors’ original file for figure 1 [file 40529_2013_55_MOESM1_ESM.tif]

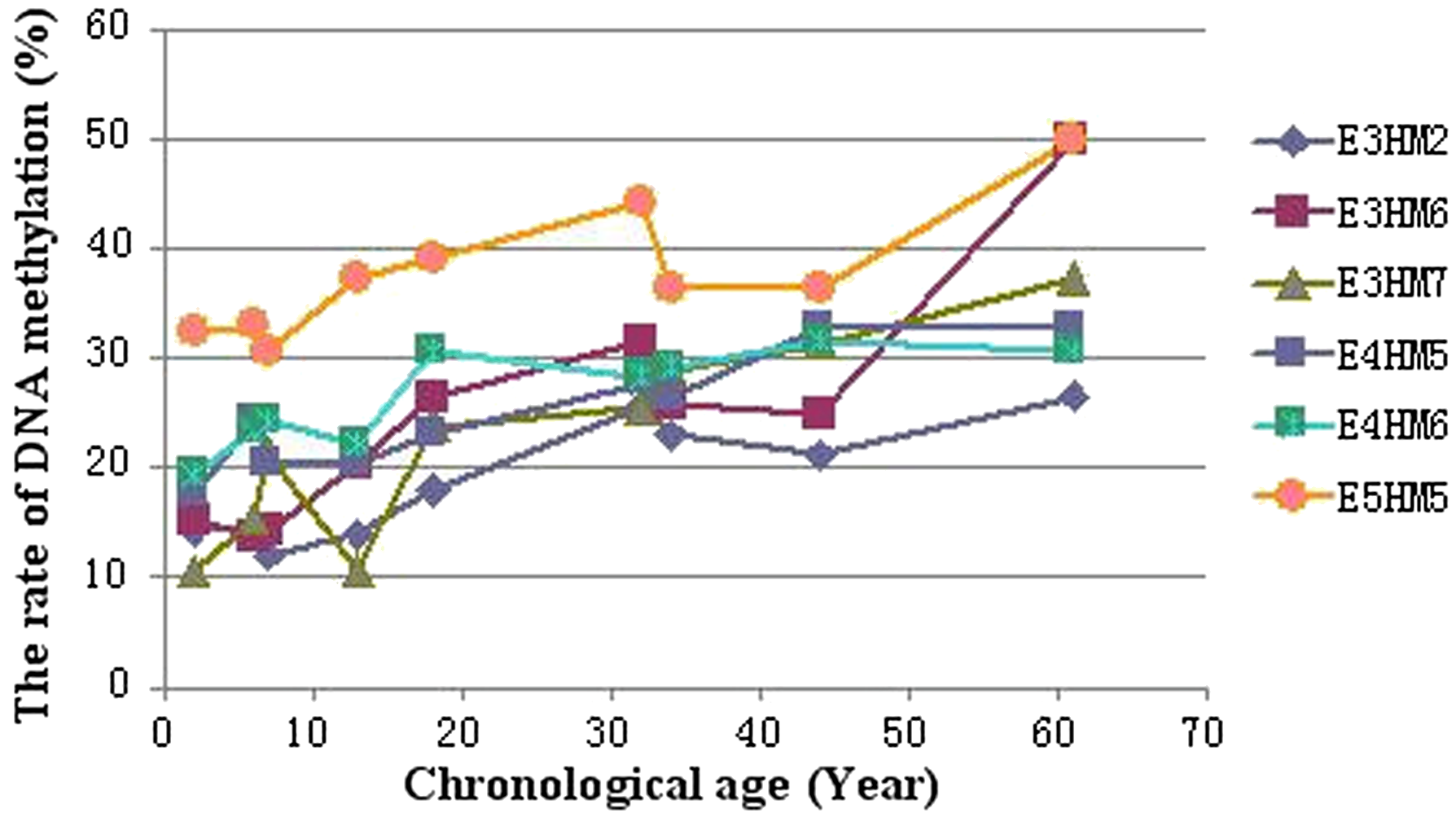

Supplement: Supplementary file 2 — Authors’ original file for figure 2 [file 40529_2013_55_MOESM2_ESM.tif]

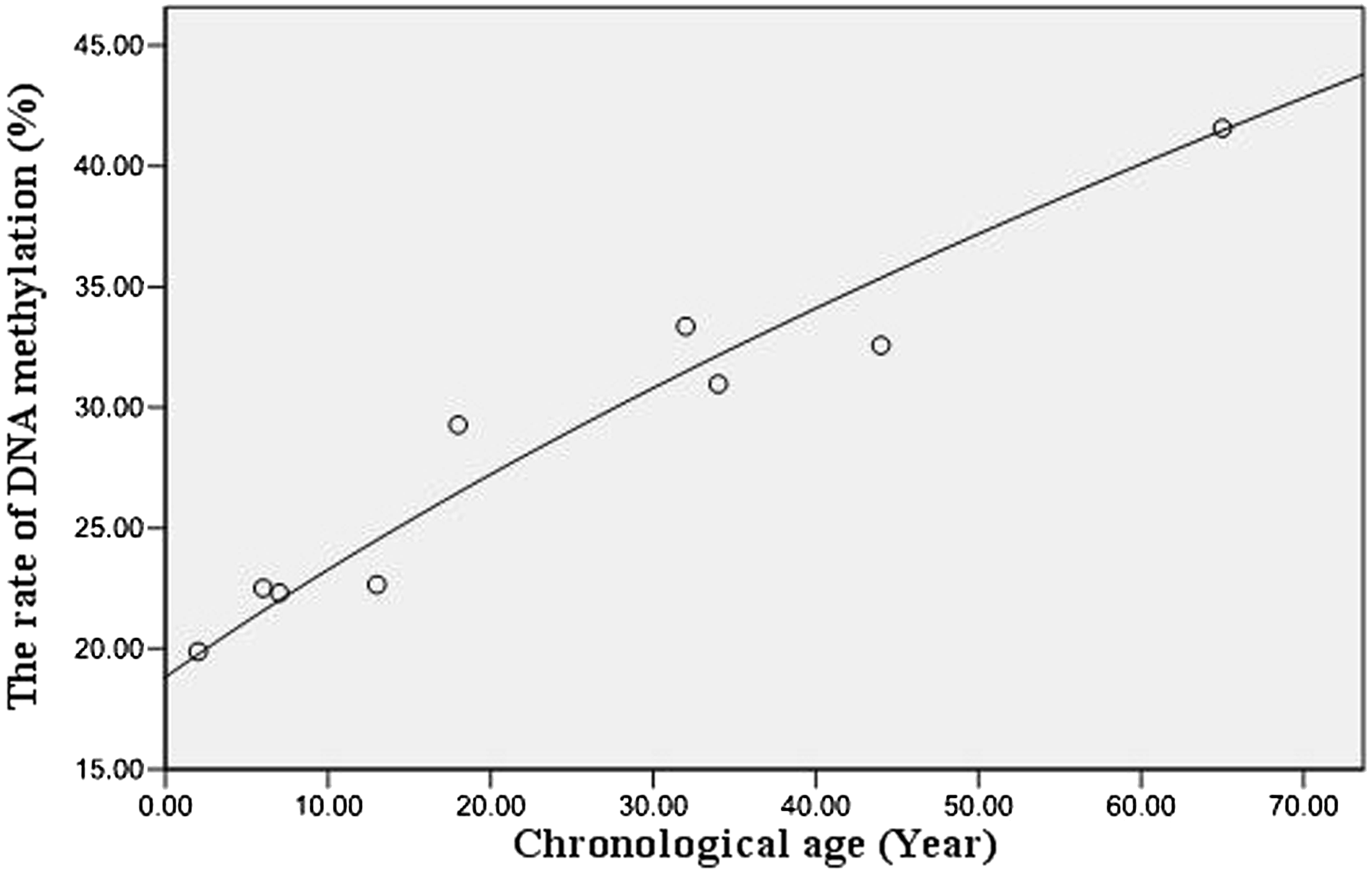

Supplement: Supplementary file 3 — Authors’ original file for figure 3 [file 40529_2013_55_MOESM3_ESM.tif]
